# Colonization with Carbapenemase producing Enterobacterales (CPE) and associated alteration in microbiota composition in a tertiary care hospital in Egypt

**DOI:** 10.1186/s12866-025-04575-3

**Published:** 2026-01-27

**Authors:** Inas El-Defrawy, Manar Khaled, Amira El-Far, Ahmed El-Shenawy, Dalia Salem, Doaa Gamal, Nevine Fam, Hanan Ali Sayed, Noha N. Hayek, Mohamed A. Elrefaiy, Ahmed El Ray, Noha S. Soliman, May S. Soliman, Amani A. El-Kholy

**Affiliations:** 1 Microbiology Department, Theodor Bilharz Research Institute (TBRI), P.O box 12411, Giza, 30 Imbaba Egypt; 2 Public Health Department, Theodor Bilharz Research Institute (TBRI), P.O box 12411, Giza, Imbaba Egypt; 3 Hepatogastoentrology Department, Theodor Bilharz Research Institute (TBRI), P.O box 12411, Giza, Imbaba Egypt; 4 Clinical and Chemical Pathology Department, Faculty of Medicine, Cairo University, 11562, Giza, 12613 Egypt

**Keywords:** Multidrug resistant bacteria (MDR), Carbapenemase producing Enterobacterales (CPE), NDM, OXA, 48, VIM, Carbapenemases, Altered microbiota, Proteobacteria

## Abstract

**Background:**

The rising incidence of carbapenemase producing Enterobacterales (CPE)s, represents an urgent health threat due to their raised morbidity and mortality consequences. Colonization of CPE represents an important vehicle for hospital acquired infections. Microbiota dysbiosis, especially in critically ill patients, is a risk factor associated with CPE-colonized infections. In this study we aimed to characterize and assess the rate of colonization with CPE, and the possibility of subsequent infection. Also, to investigate the role of microbiota dysbiosis as a potential risk factor for colonization with CPE by comparing the gut niche in colonized patients versus non-colonized patients.

**Methods:**

Rectal swabs and stools were collected from 70 patients attending Hepatogastroentrology Department at TBRI Hospital on their first 72 h of admission and during their hospital stay. Incidence of infection with CPE was followed up in the same patients with relevant clinical specimens, mainly urine culture and sensitivity. Rectal swabs were cultured on chromogenic agar media, whereas clinical specimens were cultured following microbiological methods. Species identification and antibiotic sensitivity testing were performed using Vitek-2 compact system on suspected CPE isolates, as well as detection of carbapenemase genes by conventional PCR for detection of *bla*_NDM_, *bla*_OXA-48_, *bla*_VIM_, *bla*_KPC_ and *bla*_IMP_. The 16S rRNA profiling was used for identification of gut microbiota using Illumina Miseq Sequencing System (Illumina). EzBioCloud 16S rRNA database was used for taxonomic assignment.

**Results:**

CPE carriage was found in 22.85% (*n* = 16) of included patients. *bla*_NDM_ was found predominantly in 75% (*n* = 12) of the isolates followed by *bla*_OXA-48_ in 62.5% (10/16). *Bla*_VIM_ was found in two isolates with *bla*_NDM_ and *bla*_OXA-48,_ whereas *bla*_KPC_ and *bla*_IMP_ were not detected in all tested isolates.Urinary tract infection associated with CRE colonization was detected in 12.5% (*n* = 2). Significant predominance of Proteobacteria was found in stool of CPE carriers with *p* < 0.03.

**Conclusion:**

Our results confirm the continuous pervasiveness of carbapenem resistance in our region. Alteration in microbiota and the abundance of Proteobacteria in CPE carriers may indicate a predisposition to inflammatory states in those patients and the requirement to further studies on the associated health effect. Efficient antimicrobial stewardships, strict infection control measures, and active surveillance programs are mandatory to limit the spread and subsequent infection with CPE isolates.

**Supplementary Information:**

The online version contains supplementary material available at 10.1186/s12866-025-04575-3.

## Background

Carbapenems are considered as the last therapeutic options for treatment of multidrug resistant (MDR) Gram-negative bacterial infections [1]. The rising incidence of carbapenem-resistant Enterobacterales (CRE), usually in MDR pathogens, represents an urgent health threat due to their morbidity and mortality consequences that could exceed 50% in critically ill patients. Thus, the World Health Organization (WHO) reported CRE as critical priority pathogens highlighting the urgency for active surveillance and prompt containment strategies [2]***.*** Carbapenemase production is the main mechanism of resistance in CRE. Other mechanisms of resistance include overproduction of AmpC or extended-spectrum β-lactamase (ESBL) in combination with impermeability of outer membrane porin and active efflux mechanisms [3]***.*** While CRE are known for their therapeutic challenges, carbapenemase producing Enterobacterales (CPE)s, defined with a detectable carbapenemase gene, pose a significant threat for both treatment and infection control, as carbapenemase genes are usually carried on transferrable plasmids leading to hospital outbreaks [4]. Carbapenemase production usually results in clinically relevant levels of carbapenem resistance, however it may only result in reduced susceptibility that does not reach the susceptibility breakpoints. That makes the detection of CPEs more challenging and of paramount importance [1, 2, 4].

According to Ambler classification of beta lactamases, carbapenemases are located into the three classes (A, B, and D). Among these, KPC (class A), NDM, VIM and IMP (class B; metallo-beta-lactamases (MBL)s), and OXA-48-like (class D) are the most clinically significant carbapenemases in Enterobacterales [2]. KPC and OXA-48 both utilize serine residues in their activity [4].

Colonization of CPE represents an important precondition for subsequent hospital acquired infections whether among healthcare workers or patients**.** There are multiple risk factors associated with CPE colonized infections, such as prior antimicrobial exposures, prolong hospitalization, comorbidities, the presence of invasive medical devices, as well as microbiota dysbiosis especially in critically ill patients [2, 5, 6]***.*** The abundant phyla in the gut microbiome normally are Firmicutes (60–75%), Bacteroidetes (30–40%), Actinobacteria, and Proteobacteria. This normal microbiota composition protects against the enteric pathogens, including MDRs, and reduce the colonization pressure. However, microbiota disrupting exposures such as prior antibiotics intake compromises colonization resistance, thus facilitating CRE persistence and overgrowth [7]. The 16S rRNA profiling has been used for identification of gut microbiota yielding sufficient insight about their composition and function [5].

In this study we aimed to characterize and assess the rate of community and hospital acquired colonization with CPE, and the possibility of subsequent infection. Also, to investigate the role of microbiota dysbiosis as a potential risk factor for colonization with CPE by comparing the gut niche in colonized patients versus non-colonized patients.

## Methods

### Patients and specimens included

Rectal swabs were collected from 70 patients attending Hepatogastroentrology Department at TBRI Hospital on their first 72 h of admission.

History taken from participants included education background, comorbidities (diabetes mellitus, hypertension, chronic liver disease and previous history of inflammatory bowel disease), the usage of medical devices, previous hospitalization and previous administration of antibiotics.

CPE cases were defined as adult patients with CPE intestinal colonization identified by rectal swab screening culture within 72 h of admission to the TBRI hospital. The patients who were found negative at admission were monitored by rectal swabs and stool specimens during their hospital stay by repeating the specimens on weekly basis until turned positive or being discharged from the hospital. DNA extraction from fecal specimens obtained from patients was performed for later 16S rRNA sequencing.

As reported in previous studies [8, 9], community and hospital colonization were categorized according to the timing of positive rectal swabs, so colonization detected later in patients whose admission screening was negative for CPE was classified as hospital colonization.

Incidence of infection with CPE was followed up in the same patients with relevant clinical specimens, mainly urine culture and sensitivity.

Patients were excluded from the study participation if they were under 18 years old, had a history of travelling outside Egypt in the past three months, suffered from acute bacterial or viral or active inflammatory bowel disease within 14 days before sample collection, fecal incontinence device or colostomy at the time of sample collection. These exclusion criteria were applied to reduce potential confounding effects of colonic inflammation and ensure that samples will be representative of recently passed stool. Patients participated in the study only for once.

### Microbiological cultural methods

Rectal swabs were screened for CPE by culture on CHROM ID CARBA SMART agar (BioMérieux, France). This is a ready-made biseptate chromogenic agar plate (CARB and OXA sides) of two different types of media with mixture of antibiotics which enables the selective growth of CPEs mainly of KPC and metallo-beta-carbapenemase-type on the CARB medium side, and OXA-48-type on the other OXA side.

The cultured plates were then incubated aerobically at 37 °C to be inspected for growth after 18–24 h.

Colonies on CHROM ID CARBA SMART were inspected for their color and interpreted provisionally as manufacturer’s instructions; *E. coli* (pink to burgundy color), *Klebsiella*, *Enterobacter*, *Serratia* and *Citrobacter* (bluish-green to bluish-grey or purple color).

Each type of colonies was further subcultured on MacConkey’s agar and blood agar for further species confirmation and antibiotic sensitivity testing (AST).

### Species confirmation and antibiotic sensitivity testing (AST)

Identification to the species level and AST of all CPE isolates were performed by Vitek-2 compact system, where sensitivity was performed and interpreted according to CLSI, 2024 breakpoints to the two carbapenems: meropenem (MEM) (sensitive (S) ≤ 1 μg/ml) and imipenem (IPM) (S ≤ 1 μg/ml), as well as to ceftazidime (CAZ) (S ≤ 4 μg/ml), cefepime (FEP) (S ≤ 2 μg/ml), piperacillin (PRL) (S ≤ 8 μg/ml), piperacillin/tazobactam (TZP) (S ≤ 8/4 μg/ml), ticarcillin/clavulanic acid (TCC) (S ≤ 16/2 μg/ml), aztreonam (ATM) (S ≤ 4 μg/ml) as well as ciprofloxacin (CIP) (S ≤ 0.25 μg/ml), minocycline (MIN) (S ≤ 4 μg/ml), amikacin (AK) (S ≤ 4 μg/ml), tobramycin (TOB) (S ≤ 2 μg/ml), gentamycin (CN) (S ≤ 2 μg/ml) and trimethoprim/sulfamethoxazole (SXT) (S ≤ 2/38 μg/ml) [10]. Epidemiological cut-off points “ECOFFs” released by the European Committee on Antimicrobial Susceptibility Testing (EUCAST) were also referred to in cases of isolates recovered on chromogenic agar media and showing sensitivity to carbapenems, where the screening cut-off breakpoint of meropenem was much reduced (R > 0.125 μg/ml), [11, 12].

### Polymerase chain reaction (PCR) for carbapenemase genes

DNA extraction and detection of carbapenemase genes were performed to all recovered Enterobacterales by conventional PCR using previously described primers for detection of *bla*_NDM_, *bla*_OXA-48_, *bla*_VIM_, *bla*_KPC_ and *bla*_IMP_ by Poirel et al. in 2011 [13]. PCR conditions included initial denaturation at 94 °C for 10 min, followed by 34 cycles of amplification (94 °C for 30 s, 52 °C for 40 s and 72 °C for 50 s) and lastly a final extension step at 72 °C for 5 min. Detection of amplified PCR products was performed on 2% agarose gel electrophoresis.

### Sequencing for 16Sr RNA

Stool specimens were kept at −70 °C till DNA extraction.

Stool DNA extraction of 13 CPE carriers and 20 non-carriers was performed using QIAamp Fast DNA stool mini kits (Qiagen) in accordance with the manufacturer’s instructions. Primers 341 F and 805R that target bacterial 16S rRNA gene were used for bacterial PCR amplification. The 16S rRNA gene (V4 region) was addressed utilizing universal primers that target a broad range of bacteria (F: 50-GTGCCAGCMGCCGCGGTAA-30, R: 50-GGACTACHVGGGTWTCTAAT −30), incorporating a unique 6-bp barcode for each sample associated with the paired primer [14]. The amplified products were purified and sequenced with Illumina Miseq Sequencing System (Illumina). By using the EzBioCloud 16S rRNA database, raw sequencing data was quality filtered and subjected to rarefaction analysis to evaluate sequencing depth, followed by further taxonomic classification and annotation of operation taxonomic units [15].

The microbiota composition was categorized at the phylum, genus, and species taxonomical levels, and was represented as the average relative abundance (RA). The assessment of Alpha and Beta microbial diversities was conducted using the Shannon index for Alpha diversity and the Bray–Curtis dissimilarity index for Beta diversity. Linear discriminant analysis effect size (LEfSe) was employed to identify the distinctive taxonomic biomarkers between the CRE/CPE positive (+ ve) and CRE/CPE negative (-ve) groups. Ultimately, group comparisons were made between CRE/CPE positive and negative groups in terms of microbial taxonomical composition as well as alpha and beta diversities.

### Statistical analysis

Data were entered and managed using IBM SPSS version 25 and JAMOVI software (version 2.3.28). The association between categorical variables was primarily examined using Chi-square test of independence for nominal data. When the expected count in any cell was less than five, Fisher's exact test was used to determine the exact P-value. The strength of association was measured using the Odds Ratio (OR) with a 95% Confidence Interval (CI). For reliability, a Haldane correction was applied to contingency tables where any cell count was zero. Variables that showed no detection in any CPE positive patients were excluded from the analysis. To facilitate the calculation of interpretable positive odds ratios, certain variables were re-categorized or mathematically transformed. Statistical significance was defined as a *P* value w below *P* < 0.05).

Further analyses were conducted using the EzBiocloud software (EzBiome, Inc, USA). The Wilcoxon rank-sum test was employed to assess significant differences in microbial abundance and alpha diversity across group comparisons. Additionally, PERMANOVA (per-mutational multivariate analysis of variance) was utilized to evaluate the differences in beta diversity measures among the study groups.

## Results

Between March 2022 and September 2022, 70 patients with equal sex distribution were included in the study. The median age of the participants was 56.6 years (min 19 – max 77). Sixteen patients (10 females and 6 males) were identified as CPE carriers (22.85%), and 54 (77.14%) were non carriers by culture results on chromogenic agar media. Thirteen were positive at admission and three acquired CPE during their hospital stay as detected in their follow up rectal swab cultures (3/16; 18.75%). Prior hospitalization was found in five of the CPE negative patients who were administered to the ICU. Unfortunately, history of previous administration of antibiotics was not conclusive as nearly all patients were exposed to several antimicrobial agents, due to the irrational use of antibiotics available over the counter, other risk factors were not significant (Table 1).

**Table 1:** Risk factors for CPE colonization

Risk factor	CPE		Total	Odds ratio	Confidence interval	*P* value
	**Positive**	**Negative**				
Age				1.015	0.243- 4.247	1
< 40	3 (4.3%)	10 (14.3%)	13 (18.6%)			
> 40	13 (18.6%)	44 (62.9%)	57 (81.4%)			
Sex				1.933	0.615- 6.074	0.255
Female	10 (14.3%)	25 (35.7%)	35 (50%)			
Male	6 (8.6%)	29 (41.4%)	35 (50%)			
Education				3.837	0.456—32.284	0.272
non-educated	15 (21.4%)	43 (61.4%)	58 (82.9%)			
educated	1 (1.4%)	11 (15.7%)	12 (17.1%)			
Occupation				5.21	0.282–96.3	0.339
working	0 (0%)	7 (10.0%)	7 (10.0%)			
not-working	16 (22.9%)	47 (67.1%)	63 (90.0%)			
Diabetes mellitus				9.71	0.543–173	0.055
negative	16 (22.9%)	42 (60.0%)	58 (82.9%)			
positive	0 (0.0%)	12 (17.1%)	12 (17.1%)			
Hypertension				1.322	0.426 −4.100	0.628
positive	7 (10.0%)	20 (28.6%)	27 (38.6%)			
negative	9 (12.9%)	34 (48.6%)	43 (61.4%)			
Chronic liver disease				1.154	0.321–4.145	1
negative	12 (17.1%)	39 (55.7%)	51 (72.9%)			
positive	4 (5.7%)	15 (21.4%)	19 (27.1%)			
Chronic renal disease				10.5	0.409 −272	0.229
positive	1 (1.4%)	0 (0.0%)	1 (1.4%)			
negative	15 (21.4%)	54 (77.1%)	69 (98.6%)			
History of chemotherapy				2.94	0.150–57.6	0.567
negative	16 (22.9%)	50 (71.4%)	66(94.3%)			
positive	0 (0.0%)	4 (5.7%)	4 (5.7%)			
History of antibiotic use				1.733	0.147–20.456	0.547
positive	1 (1.4%)	2 (2.9%)	3 (4.3%)			
negative	15(21.4%)	52 (74.3%)	67(95.7%)			
History of ICU				3.67	0.192–69.9	0.582
positive	0 (0.0%)	5 (7.1%)	5 (7.1%)			
negative	16 (22.9%)	49 (70.0%)	65 (92.9%)			

**Fig. 1:**
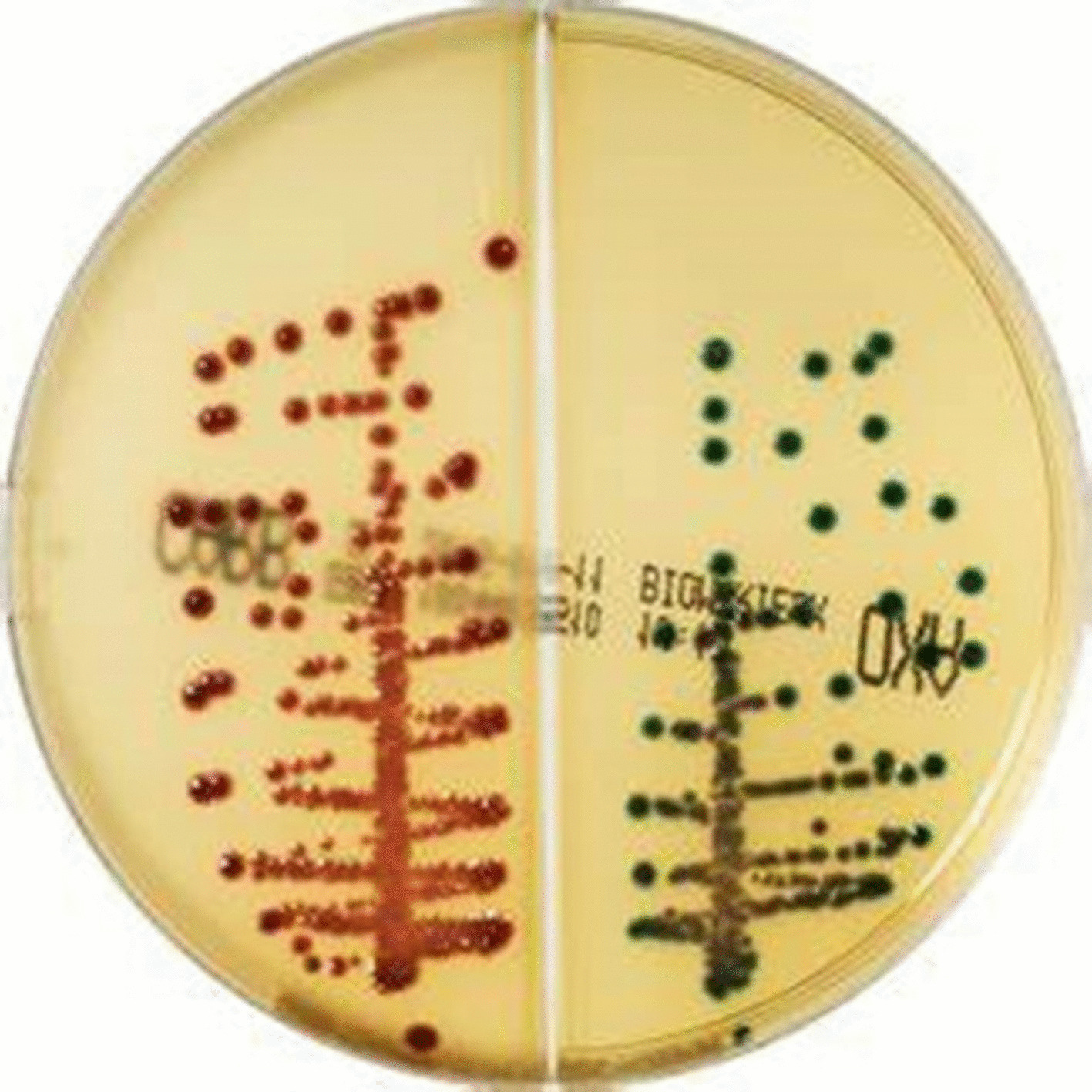
CHROM ID CARBA SMART agar showing *E. coli* pink colonies on CARB side and *Klebsiella pneumoniae* blue colonies on OXA side

CPE isolates included *E. coli* as the most commonly detected species (9/16; 56.25%), followed by *K. pneumoniae* (7/16; 43.75%) as identified by their colony color in accordance with the manufacturer’s documents of CHROM ID CARBA SMART agar (Fig. 1). Confirmation of species identification and antibiotic sensitivity testing was performed by Vitek-2 system.

Antibiotic sensitivity testing by Vitek-2 compact system, following CLSI, 2024 breakpoints, showed that 11 CPE isolates were resistant to both carbapenems (imipenem and meropenem). Whereas two isolates were sensitive to both carbapenems (Imipenem; MIC = 0.5 μg/ml, meropenem; MIC = 0.5 & 1 μg/ml). three isolates were found intermediate to imipenem (MIC = 2 μg/ml), one of them was also intermediate to meropenem (MIC = 2 μg/ml) and the other two were sensitive to meropenem (MIC ≤ 0.25 & 1 μg/ml), however they were considered resistant by ECOFFs. (Table 2). Regarding β-lactam antibiotics; all 16 CPE isolates were resistant to ceftazidime, cefepime, and β-lactam/β-lactamase inhibitor combinations, including piperacillin/tazobactam, except one *bla*_OXA-48_ positive *E. coli* isolate that was sensitive to ceftazidime and cefepime (MIC = 1 μg/ml for both), whereas 12.5% (2/16) were sensitive to aztreonam. Best sensitivity was found to aminoglycosides primarily to amikacin (10/16; 62.5%), followed by gentamicin (8/16; 50%), then tobramycin (5/16; 31.25%). Sensitivity to ciprofloxacin was 12.5% (2/16), and to minocycline 18.75% (3/16). All isolates were resistant to trimethoprim/sulfamethoxazole.

**Table 2:** Antibiotic sensitivity testing results to carbapenems of 16 CPE isolates recovered on chromogenic agar media in relation to their carbapenemase genes

Isolate	Species	Carbapenemase producing genes detected by PCR	Sensitivity to carbapenems (MIC(μg/ml); Interpretation(S/I/R)*	
			**Imipenem**	**Meropenem**
**6R**	*E. coli*	*bla*_NDM_, *bla*_OXA-48_, *bla*_VIM_	≥ 16; R	8; R
**8R**	*K. pneumonia*	*bla*_NDM_, *bla*_OXA-48_	≥ 16; R	≥ 16; R
**13R**	*E. coli*	*bla*_NDM_	≥ 16; R	≥ 16; R
**26R**	*E. coli*	*bla*_NDM_	≥ 16; R	≥ 16; R
**27R**	*E. coli*	*bla*_OXA-48_	0.5; S	1; S
**31R**	*K. pneumonia*	*bla*_NDM_	≥ 16; R	≥ 16; R
**42R**	*E. coli*	*bla*_NDM_, *bla*_OXA-48_	≥ 16; R	≥ 16; R
**47R**	*E. coli*	*bla*_NDM_, *bla*_OXA-48_	≥ 16; R	≥ 16; R
**52R**	*E. coli*	*bla*_NDM_, *bla*_OXA-48_	≥ 16; R	≥ 16; R
**60R**	*E. coli*	*bla*_OXA-48_	0.5; S	0.5; S
**61R**	*K. pneumonia*	*bla*_OXA-48_	2; I	≤ 0.25; S
**69R**	*K. pneumonia*	*bla*_NDM_, *bla*_OXA-48_, *bla*_VIM_	2; I	2; I
**71R**	*K. pneumonia*	*bla*_NDM_	≥ 16; R	≥ 16; R
**72R**	*E. coli*	*bla*_OXA-48_	2; I	1; S
**73R**	*K. pneumonia*	*bla*_NDM_	≥ 16; R	8; R
**74R**	*K. pneumonia*	*bla*_NDM_	≥ 16; R	≥ 16; R

^*^MIC Minimum inhibitory concentration, *S* Sensitive, *I* Intermediate, *R* Resistant

Carbapenemase genes were detected in all tested isolates confirming they were all CPEs. According to PCR results four genotypes of carbapenemases genes existed (Table 3); where *bla*_NDM_ was found alone in six isolates (four *K. pneumoniae* and two *E. coli*), and with *bla*_OXA-48_ in four isolates (three *E. coli* and one *K. pneumoniae*). Two isolates (one *E. coli* and one *K. pneumoniae*) had the three genes (*bla*_NDM_, *bla*_OXA-48_ and *bla*_VIM_), *bla*_OXA-48_ existed alone in four isolates (three *E. coli* and one *K. pneumoniae*). So *bla*_NDM_ was the predominant gene in (12/16) (75%) of all CPE isolates; mainly in *K. pneumoniae* isolates (*n* = 6/7) (85.7%), followed by *bla*_OXA-48_ in 62.5% (10/16), predominantly in *E. coli* isolates (*n* = 7/9) (77.8%). Two isolates had *bla*_VIM_ together with *bla*_NDM_ and *bla*_OXA-48,_ whereas *bla*_KPC_ and *bla*_IMP_ were not detected in all tested isolates.

**Table 3:** Types, number and distribution of resistance genes among isolated species from 16 CPE colonized patients

Type of Resistance genes	Number of Isolates	Number of isolates in each species	
		***K. pneumoniae***	***E. coli***
1- *bla*_NDM_	6	4	2
2- *bla*_OXA-48_	4	1	3
3- *bla*_NDM_ + *bla*_OXA-48_	4	1	3
4- *bla*_NDM_ + *bla*_OXA-48_ + *bla*_VIM_	2	1	1
**Total**	16	7	9

Urinary tract infection was confirmed in two out of the 16 CPE carriers (12.5% (2/16)), one at admission and the other was detected with the follow up clinical specimens. Isolates were of the same species and molecular characterization as the colonizing CPE ones; one was *E. coli* and the other was *K. pnuemoniae* and both were positive for *bla*_NDM_ only.

**Fig. 2:**
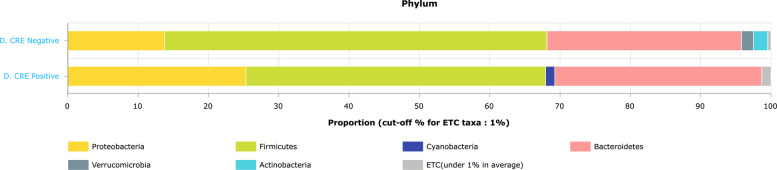
EzBioCloud analysis showing relative abundance of composition of different phyla existing in CRE negative versus CRE positive isolates

**Fig. 3:**
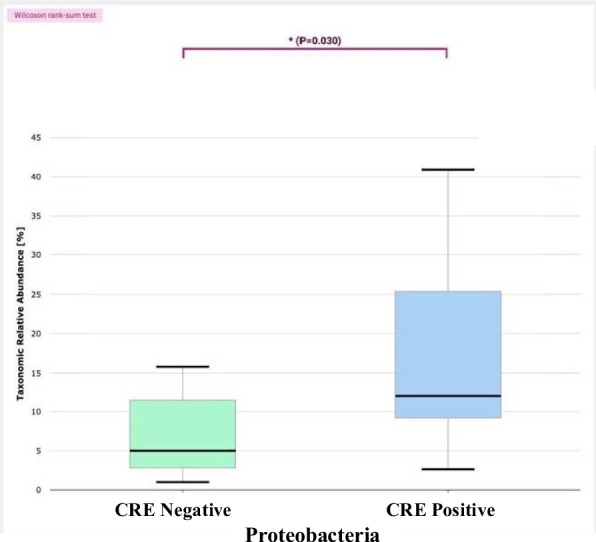
Boxplot showing significant increased proportion of Proteobacteria in gut microbiota in CRE carriers compared to CRE non-carriers (Wilcoxon rank-sum test, *p* = 0.03). The boxes illustrate interquartile ranges (IQR), with the horizontal line indicating the median and whiskers extending to the most extreme values

**Fig. 4:**
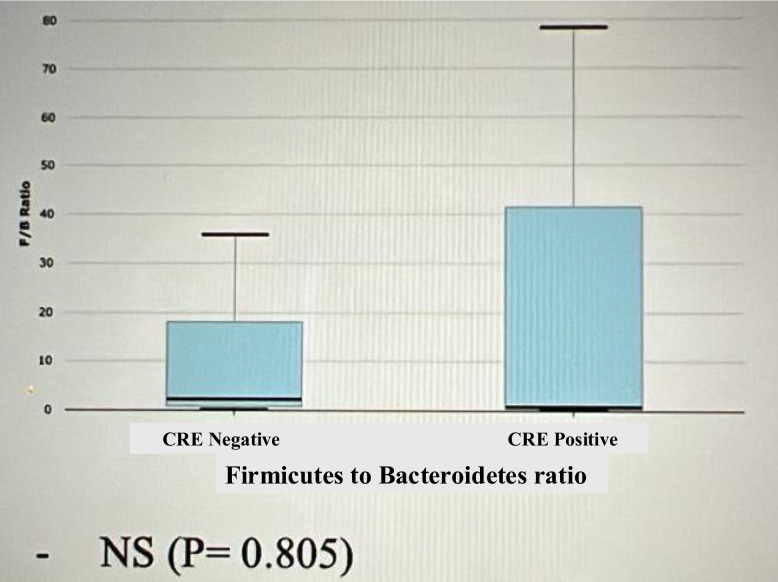
Microbiota taxonomical analysis showing non-significance decrease in Fermicutes/Bacteriodetes (F/B) ratio in CRE positive relative to CRE negative isolates (*p* = 0.796)

**Fig. 5:**
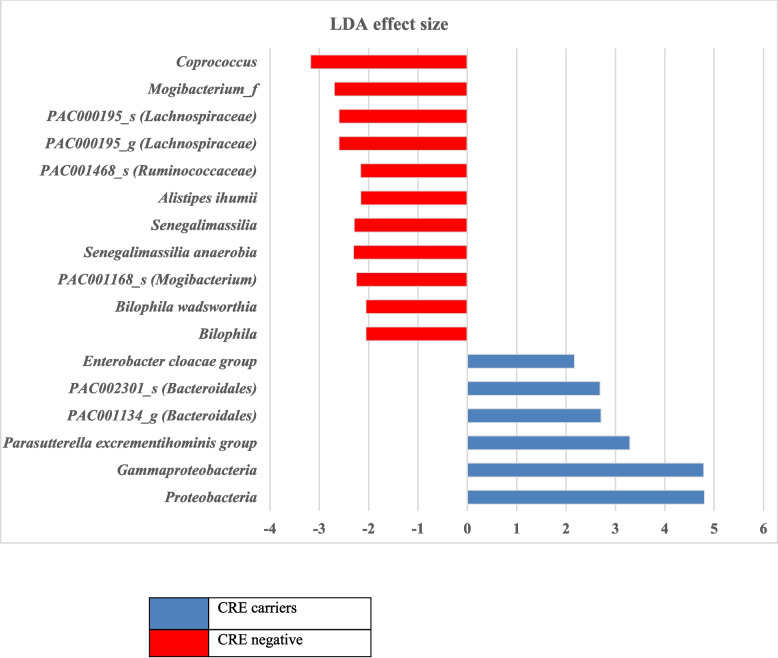
Linear discriminant analysis (LDA) scores obtained from linear discriminant effect size (LEfSe) analysis of fecal microbiome. An LDA effect size of more than 2 was used as a threshold for the LEfSe analysis showing the top genera in CRE carriers; *Enterobacter cloacae*, Bacteroidales, *Prasutterella excrementihominis*, Gammaproteobacteria, Proteobacteria. Whereas other genera as Coprococcus, Mogibacterium, Bilophilia showed lower 2 LDA scores in CRE negative patients

Using the EzBioCloud 16S rRNA database for taxonomic assignment comparative MTP analyzer of stool microbiota between 20 CPE negative stool specimens and 13 CPE positive revealed the following:

The relative abundance of the phyla in CPE carriers comprised 43% Firmicutes, 29% Bacteroidetes and 25% Proteobacteria (Fig. 2). In CPE non-carriers, the relative abundance of the phyla comprised 54% Firmicutes, 28% Bacteroidetes and 14% Proteobacteria. Thus, gut microbiota from CPE carriers showed a significant increased proportion of Proteobacteria (*p* = 0.03), (Fig. 3). In addition, reduction in Fermicutes/Bacteriodetes (F/B) ratio was noticed, though not statistically significant (*p* = 0.796) (Fig. 4). Also, Linear discriminant analysis (LDA) with linear discriminant effect size (LEfSe) revealed that several bacterial genera were noticeably altered in the gut microbiota of CPE carriers compared with those in non-carriers. Among CPE carriers, the top genera were *Enterobacter cloacae*, *Bacteroidales*, *Prasutterella excrementihominis*, *Gammaproteobacteria* and *Proteobacteria*, whereas other genera as *Coprococcus*, *Mogibacterium*, *Bilophilia* showed lower 2 LDA scores (Fig. 5).

**Fig. 6:**
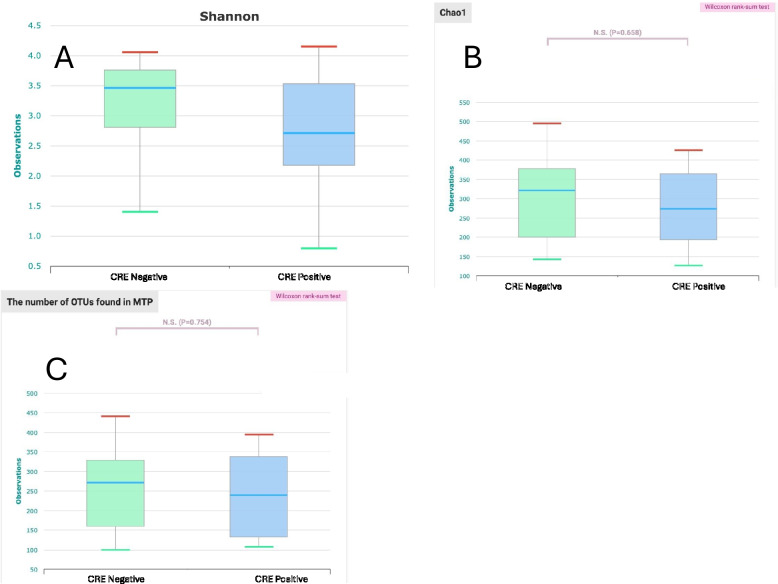
Gut Microbial Alpha diversity indices among CRE positive and negative groups showing non-significant decrease in CRE carriers analyzed by Wilcoxon rank-sum test: A) Shannon, B) Chao1, C) number of OTUs

**Fig. 7:**
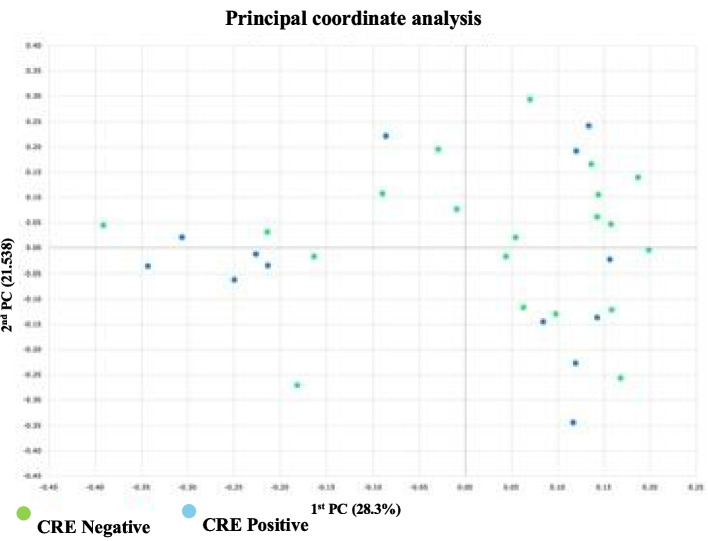
Beta diversity analysis of Gut microbiota using Bray–Curtis dissimilarity index. among CRE positive and negative groups, where the 2 groups are not significantly apart (*p* = 0.178). A principal coordinate analysis (PCoA) plot illustrating the degree of similarity in microbiota profiles between the two groups, with each coordinate indicating the percentage of variance. The differences between study groups were assessed by PERMANOVA test (per-mutational multivariate analysis of variance)

The alpha diversity indices (Shannon, Chao1, and OTUs) were non-significantly decreased inCPE carriers compared to the CPE non-carriers (*p* = 0.210, 0.658, 0.754 respectively) (Fig. 6). However, the beta diversity analysis (Bray–Curtis distances) demonstrated that the two groups were not significantly apart (*p* = 0.178) (Fig. 7).

## Discussion

Intestinal colonization with CPE poses a significant source of community dissemination and hospital acquired infection. Therefore, it is mandatory to promptly identify and isolate CPE carriers to reduce transmission and improve patients’ outcomes [12].

The current work aimed to assess the rate of colonization of CPE, the possibility of subsequent infection, and the associated alteration in the gut microbiota.

Our results showed that CPE carriage was found in 22.85%, which is comparable with previous studies in Egypt, where it represented 24% & 28% [12, 16]***.*** Lower rates of colonization were obtained from Kuwait that ranges between 8 to 12% [17], 17% in Nigeria [18], and 9.6% in Kenya (9.6%) [19]. Colonization prevalence of CPE is reported highest in Asia and Africa, whereas lower, but still significant, rates were detected in the Americas and Europe. This geographical variations in colonization prevalence confirms the need to identify contributing regional factors in the community, including poor water and sanitation [20].

In this study colonization that occurred after 72 h from admission was considered hospital acquired and it represented 19% of total carriers that was going with what has been reported recently about the three days median duration suggested from admission to colonization with CPE. However, a limitation in the current work is that it represents a single center study that may reflect the environment of our hospital, located in an endemic area with poor sanitary resources. Thus, though most of the CPE colonization was found to be community acquired, that may represent the special nature of the patients attending our hospital who were mainly from the neighborhood. It is also suggested that variation in colonization rates occur within different departments and could be associated with pharmacological therapies, patient groups and clinical settings [21].

No significant differences were found between CPE positive and negative groups regarding risk factors, as the presence of comorbidities, invasive procedures, previous administration of antibiotics and previous ICU admission (all *P* > 0.05). This was in accordance with previous single center study that could not identify any clinical risk factors which were significantly associated with acquisition of gut pathogens [9]. However, that may be attributed to the endemic location of our hospital and the small sample size included in the current study.

Carbapenemases resistance genes were detected in all tested isolates confirming it was the main mechanism of resistance. However, according to CLSI, 2024 breakpoints, five isolates were not resistant, where *bla*_OXA-48_ was detected in these isolates alone in four of them, and with *bla*_VIM_ and *bla*_NDM_ in one isolate (Table 2). This could be explained by the diminished carbapenemase activity of OXA-48 enzymes compared to KPC and MBLs. For this reason, detection of OXA-48-producing Enterobacterales based on susceptibility phenotype is more challenging as it may not meet the current CLSI criteria for intermediate or resistance sensitivity results [4, 10]. However, the carbapenems intermediate susceptibility results expressed in the isolate harboring the three resistance genes; *bla*_OXA-48_, *bla*_NDM_, and *bla*_VIM_ may indicate the lack of expression or the presence of uncommon enzyme derivatives (especially of NDM). Unfortunately sequencing this gene was not accessible. Resistance to other classes of antibiotics found in our study confirms the MDR nature of nearly all CPE isolates [21, 22].

Although *bla*_OXA-48_ represented the main carbapenemase producing gene in previous work [12]***,*** in the current study *bla*_NDM_ was the leading gene in 75% of the isolates, which confirms the persistent nature described of NDM carbapenemases and their tendency to be endemic in Egypt [23, 24]. *Enterobacterales* producing OXA-48 are endemic in other neighboring countries as Tunisia, Morocco and [25]. Rectal swabs surveillance specimens from ICU patients in Bahrain, Kuwait, Saudi Arabia, Oman, and the United Arab Emirates in 2019 revealed *bla*_OXA-48_ as the most prevalent carbapenemase gene in 15% of all specimens screened and up to 51% of those in Saudi Arabia [25, 26]. Different regions in the world and sometimes difference in same countries show different predominance in CPE determinants [12]. The rise in carbapenem resistance was reported globally by the European Center for Disease Prevention and Control (ECDC) and the World Health Organization (WHO) where the prevalence of carbapenem resistance in *K. pneumoniae* isolates raised by 20% in 2023 compared to 2017,whereas the national average of resistance to carbapenems in *E. coli* remained at 1.6% [27]**.** The overall risk of CPE infection in colonized patients was reported as 16.5% and 22.5% in other studies [28, 29]***.*** This is comparable with our study where infection associated with CPE colonization was detected in 12.5%, thus supporting the strong association between CPE colonization and development of clinical infections previously reported [1].

Normal constituent of Microbiota in the Egyptian population is still unrevealed and needs more advanced future studies to correlate types of dysbiosis due to CPE carriage. Proteobacteria represent one of the most abundant phyla in the human gut microbiota and its dominance is associated with increased inflammation. It was reported that CPE carriers have higher number of Proteobacteria and lower prevalence of Firmicutes and Bacteroidetes than non-carriers and healthy controls [30].

Our results confirmed the significant predominance of Proteobacteria in CPE carriers with *p* < 0.03. However, the decrease in Bacteroidetes phyla was not significant. The Enterobacterales family are normally present in small fraction of total bacteria in healthy individuals, whereas it can abnormally proliferate in the gut of individuals affected by intestinal or extra-intestinal diseases such as obesity, inflammatory bowel disease or metabolic disorders [31]. It has been reported that Proteobacteria abundance is implicated in the genesis of endotoxemia and in the development of metabolic disorders [32].

Similarly, LDA with LEfSe showed alteration in several bacterial genera in the gut microbiota of CPE carriers, though not statistically significant, compared with those in non-carriers. Although as reported by *Baek *et al*., 2*023 [5], the alpha diversity was decreased in CPE carriers compared to the CPE non-carriers, however Shannon index was statistically non-significantly (*p* = 0.210), also the beta diversity analysis (Bray–Curtis distances) demonstrated that the two groups were not significantly apart (*p* = 0.178). Further studies of larger sample sizes may reveal more details in that context.

Our study has limitations that included being a single center study, the small sample size that hindered the performance of multivariate analysis for risk factors as they were not statistically significant.

## Conclusion

Increased laboratory capacity to improve and shorten duration of detection is on top priorities to combat challenge of antimicrobial resistance. Detection of CPE colonization, efficient antimicrobial stewardships, strict infection control measures and active surveillance programs are imperative to trace and prevent their acquisition and spread. The use of differential chromogenic agar in screening in endemic regions, as ours, is highly recommended. Alternatively, the application of ECOFFs is encouraged to screen to detect low enzymatic activity of some carbapenemases as OXA-48-like. Our results confirm the pervasiveness of carbapenem resistance in our region especially NDM enzyme. Alteration in microbiota and the abundance of Proteobacteria in CPE carriers may indicate a predisposition to inflammatory states. Further multi-centered studies of larger sample size are needed to investigate the effect of microbiota alteration on the health of patients colonized by CPE. 

## Supplementary Information

Supplementary Material 1

## Data Availability

BioProject: PRJNA1364764: Colonization with Carbapenemase producing Enterobacterales (CPE) and Microbiota. All data generated or analyzed during this study are available at [https://www.ncbi.nlm.nih.gov/sra/PRJNA1364764] (https://www.ncbi.nlm.nih.gov/sra/PRJNA1364764). All data generated or analyzed during this study are available at https://www.ncbi.nlm.nih.gov/sra/PRJNA1364764.

## Abbreviations

AST: Antibiotic sensitivity test
CPE: Carbapenemase producing Enterobacterales
CRE: Carbapenem resistant Enterobacterales
*E. coli*: *Escherichia coli*
ECDC: European Center for Disease Prevention and Control
ECOFFS: Epidemiological cut-off points
ESBL: Extended-spectrum-ꞵ-lactamase
EUCAST: European Committee on Antimicrobial Susceptibility Testing
K. pneumoniae: Klebsiella pneumoniae
MBL: Metallo-beta-lactamases
MDR: Multidrug resistant bacteria
MIC: Minimal inhibitory concentration
SPSS: Statistical Package for Social Sciences
TBRI: Theodor Bilharz Research Institute
WHO: World Health Organization

## References

[1] Hassoun-Kheir N, Hussien K, Karram M, et al. Clinical significance and burden of carbapenem-resistant Enterobacterales (CRE) colonization acquisition in hospitalized patients. Antimicrob Resist Infect Control. 2023;12:129. 10.1186/s13756-023-01323-y. 37986092 10.1186/s13756-023-01323-y PMC10658805

[2] Alvisi G, Curtoni A, Fonnesu R, et al. Epidemiology and genetic traits of carbapenemase-producing Enterobacterales: a global threat to human health. Antibiotics. 2025;14(2):141. 10.3390/antibiotics14020141. 40001385 10.3390/antibiotics14020141 PMC11852015

[3] Li Y, Mai Y, Liu Y, Jiang Y. Epidemiological characteristics and carbapenemase analysis of carbapenem-resistant enterobacterales isolates in a teaching hospital in Guangzhou China. Infect Drug Resist. 2025;18:2105–17. 10.2147/IDR.S507692. 40303604 10.2147/IDR.S507692 PMC12039840

[4] Iovleva A, Doi Y. Carbapenem-resistant enterobacteriaceae. Clin Lab Med. 2017;37(2):303–15. 10.1016/j.cll.2017.01.005. 28457352 10.1016/j.cll.2017.01.005 PMC5412586

[5] Baek MS, Kim S, Kim WY, Kweon MN, Huh JW. Gut microbiota alterations in critically ill patients with carbapenem-resistant Enterobacteriaceae colonization: a clinical analysis. Front Microbiol. 2023;14:1140402. 10.3389/fmicb.2023.1140402. 37082174 10.3389/fmicb.2023.1140402 PMC10110853

[6] Zheng G, Cai J, Deng H, Yang H, Xiong W, Chen E, et al. Development of a risk prediction model for subsequent infection after colonization with carbapenem-resistant Enterobacterales: a retrospective cohort study. Antimicrob Resist Infect Control. 2024;13(1):46. 10.1186/s13756-024-01394-5. 38659068 10.1186/s13756-024-01394-5 PMC11044304

[7] Lee I, Kim B, Suk KT, Lee SS. Gut microbiome-based strategies for the control of carbapenem-resistant Enterobacteriaceae. J Microbiol Biotechnol. 2025;35:1–10. 10.4014/jmb.2406.06017. 10.4014/jmb.2406.06017 PMC12351111 40774824

[8] Bastos LR, Almeida MM, Marques EA, Leão RS. Pre-operative colonization by staphylococcus aureus and cephalosporin non-susceptible bacteria in patients with proximal femoral fractures. Rev Bras Ortop (Sao Paulo). 2022;57(5):726–33. 10.1055/s-0041-1735546. 36226207 10.1055/s-0041-1735546 PMC9550373

[9] Shamalov L, Heath M, Lynch E, et al. Timing and clinical risk factors for early acquisition of gut pathogen colonization with multidrug resistant organisms in the intensive care unit. Gut Pathog. 2024;16:10. 10.1186/s13099-024-00605-z. 38383457 10.1186/s13099-024-00605-z PMC10880254

[10] Clinical and Laboratory Standards Institute Performance standards for antimicrobial susceptibility testing. M100. 34th. Wayne, PA: CLSI; 2024.

[11] European Committee on Antibiotic Susceptibility Testing (EUCAST, 2017): Testing Breakpoint Tables for Interpretation of MICs and Zone Diameters Version 5.0, Valid from 2017–01–01. Sweden: European Committee on Antibiotic Susceptibility Testing; 2017.

[12] El-Defrawy I, Gamal D, El-Gharbawy R, et al. Detection of intestinal colonization by carbapenem-resistant Enterobacteriaceae (CRE) among patients admitted to a tertiary care hospital in Egypt. Egypt J Med Hum Genet. 2022;23:83. 10.1186/s43042-022-00295-9.

[13] Poirel L, Walsh TR, Cuvillier V, Nordmann P. Multiplex PCR for detection of acquired carbapenemase genes. Diagn Microbiol Infect Dis. 2011;70(1):119–23. 10.1016/j.diagmicrobio.2010.12.002. 21398074 10.1016/j.diagmicrobio.2010.12.002

[14] Klindworth A, Pruesse E, Schweer T, Peplies J, Quast C, Horn M, et al. Evaluation of general 16S ribosomal RNA gene PCR primers for classical and next-generation sequencing-based diversity studies. Nucleic Acids Res. 2013;41:e1. 10.1093/nar/gks808. 22933715 10.1093/nar/gks808 PMC3592464

[15] Yang Y-W, et al. Use of 16S rRNA gene-targeted group-specific primers for real-time PCR analysis of predominant bacteria in mouse feces. Appl Environ Microbiol. 2015;81(19):6749–56. 10.1128/AEM.01906-15. 26187967 10.1128/AEM.01906-15 PMC4561689

[16] Ghaith DM, Mohamed ZK, Farahat MG, Aboulkasem Shahin W, Mohamed HO. Colonization of intestinal microbiota with carbapenemase-producing Enterobacteriaceae in paediatric intensive care units in Cairo Egypt. Arab J Gastroenterol. 2019;20(1):19–22. 10.1016/j.ajg.2019.01.002. 30733176 10.1016/j.ajg.2019.01.002

[17] AlFadhli AH, Jamal WY, Rotimi VO. Prevalence of carbapenem‐resistant Enterobacteriaceae and emergence of high rectal colonization rates of blaOXA‐181‐positive isolates in patients admitted to two major hospital intensive care units in Kuwait. Plos One. 2020;2020. 10.1371/journal.pone.0241971. 10.1371/journal.pone.0241971 PMC7671514 33201906

[18] Adekanmbi O, Popoola O, Fowotade A, et al. Prevalence of rectal carbapenem resistant Enterobacterales carriage among patients attending healthcare facilities in Ibadan, Nigeria: a descriptive study. BMC Infect Dis. 2024;24:726. 10.1186/s12879-024-09627-z. 39048999 10.1186/s12879-024-09627-z PMC11267743

[19] Githii S, Maingi JM, Nyaga T, Ndungu C, Nyongesa KW, Musyoki AM. Gastrointestinal carriage of carbapenemase-producing enterobacterales among inpatient and outpatient children in Kenya. Sci Rep. 2024;14(1):30684. 10.1038/s41598-024-78059-1. 39730388 10.1038/s41598-024-78059-1 PMC11680766

[20] Parra G, Lautenbach E, Mosepele M, et al. Colonization with antibiotic resistant bacteria in communities and hospitals across six countries, including Bangladesh, Botswana, Chile, Guatemala, India, and Kenya. Sci Rep. 2025;15:21275. 10.1038/s41598-025-94750-3. 40596472 10.1038/s41598-025-94750-3 PMC12218395

[21] Wang Z, Shao C, Shao J, Hao Y, Jin Y. Risk factors of carbapenem-resistant Enterobacterales intestinal colonization for subsequent infections in hematological patients: a retrospective case-control study. Front Microbiol. 2024;15:1355069. 10.3389/fmicb.2024.135506915:1355069. 38680915 10.3389/fmicb.2024.1355069 PMC11045900

[22] Gamal D, Egea P, Elías C, Fernández-Martínez M, Causse M, Pérez-Nadales E, et al. High-risk clones and novel sequence type ST4497 of Klebsiella pneumoniae clinical isolates producing different alleles of NDM-type and other carbapenemases from a single tertiary-care centre in Egypt. Int J Antimicrob Agents. 2020;56(6):106164. 10.1016/j.ijantimicag. 32949764 10.1016/j.ijantimicag.2020.106164

[23] El Defrawy I, Salem D, Ali G, et al. Carbapenemase producing Enterobacterales clinical isolates from a tertiary care hospital in Egypt. Beni-Suef Univ J Basic Appl Sci. 2023;12:98. 10.1186/s43088-023-00437-x.

[24] Gandor NHM, Amr GE, Eldin Algammal SMS, Ahmed AA. Characterization of carbapenem-resistant *K. pneumoniae* isolated from intensive care units of Zagazig University hospitals. Antibiotics. 2022;11(8):1108. 10.3390/antibiotics11081108. 36009977 10.3390/antibiotics11081108 PMC9405146

[25] Boyd SE, Holmes A, Peck R, Livermore DM, Hope W. OXA-48-like β-lactamases: global epidemiology, treatment options, and development pipeline. Antimicrob Agents Chemother. 2022;66(8):e0021622. 10.1128/aac.00216-22. (**Epub 2022 Jul 20. 10.1093/jacamr/dlac104**). 35856662 10.1128/aac.00216-22 PMC9380527

[26] Alqahtani H, Alghamdi A, Alobaidallah N, Alfayez A, Almousa R, Albagli R, et al. Evaluation of ceftazidime/avibactam for treatment of carbapenemase- producing carbapenem-resistant Enterobacterales with OXA-48 and/or NDM genes with or without combination therapy. JAC-Antimicrobial Resistance. 2022;4(5):104. 10.1093/jacamr/dlac104. 10.1093/jacamr/dlac104 PMC9552550 36237571

[27] European Centre for Disease Prevention and Control and World Health Organization. Antimicrobial Resistance Surveillance in Europe 2023–2021 Data. Available online at: https://www.ecdc.europa.eu/en/publications-data/antimicrobial-resistance-surveillance-europe-2023-2021-data. 2023

[28] Tischendorf J, de Avila RA, Safdar N. Risk of infection following colonization with carbapenem-resistant Enterobactericeae: a systematic review. Am J Infect Control. 2016;44(5):539–43. 10.1016/j.ajic. 26899297 10.1016/j.ajic.2015.12.005 PMC5262497

[29] Chu W, Hang X, Li X, Ye N, Tang W, Zhang Y, et al. Bloodstream infections in patients with rectal colonization by carbapenem-resistant enterobacteriaceae: a prospective cohort study. Infect Drug Resist. 2022;20(15):6051–63. 10.2147/IDR.S383688. 10.2147/IDR.S383688 PMC9581720 36277248

[30] Sindi AA, Alsayed SM, Abushoshah I, Bokhary DH, Tashkandy NR. Profile of the gut microbiome containing carbapenem-resistant enterobacteriaceae in ICU patients. Microorganisms. 2022;10(7):1309. 10.3390/microorganisms10071309. 35889029 10.3390/microorganisms10071309 PMC9320093

[31] de Moreira Gouveia MI, Bernalier-Donadille A, Jubelin G. Enterobacteriaceae in the human gut dynamics and ecological roles in health and disease. Biology. 2024;13(3):142. 10.3390/biology13030142. 38534413 10.3390/biology13030142 PMC10967970

[32] Rizzatti Gianenrico, Lopetuso Loris, Gibiino G, Binda Cecilia, Gasbarrini Antonio. Proteobacteria: a common factor in human diseases. Biomed Res Int. 2017. 10.1155/2017/9351507. 29230419 10.1155/2017/9351507 PMC5688358

